# The Role of Ca^2+^ Imbalance in the Induction of Acute Oxidative Stress and Cytotoxicity in Cultured Rat Cerebellar Granule Cells Challenged with Tetrabromobisphenol A

**DOI:** 10.1007/s11064-016-2075-x

**Published:** 2016-10-07

**Authors:** Elzbieta Zieminska, Jacek Lenart, Dominik Diamandakis, Jerzy W. Lazarewicz

**Affiliations:** Department of Neurochemistry, Mossakowski Medical Research Centre, Polish Academy of Sciences, Pawinskiego 5, 02-106, Warsaw, Poland

**Keywords:** Brominated flame retardants, Neuroprotection, NMDA receptors, Reactive oxygen species, Ryanodine receptors, Primary neuronal cultures

## Abstract

Using primary cultures of rat cerebellar granule cells (CGC) we examined the role of calcium transients induced by tetrabromobisphenol A (TBBPA) in triggering oxidative stress and cytotoxicity. CGC were exposed for 30 min to 10 or 25 µM TBBPA. Changes in intracellular calcium concentration ([Ca^2+^]_i_), in the production of reactive oxygen species (ROS), and in the potential of mitochondria (∆Ψm) were measured fluorometrically during the exposure. The intracellular glutathione (GSH) and catalase activity were determined after the incubation; cell viability was evaluated 24 h later. TBBPA concentration-dependently increased [Ca^2+^]_i_ and ROS production, and reduced GSH content, catalase activity, ∆Ψm and neuronal viability. The combination of NMDA and ryanodine receptor antagonists, MK-801 and bastadin 12 with ryanodine, respectively, prevented Ca^2+^ transients and partially reduced cytotoxicity induced by TBBPA at both concentrations. The antagonists also completely inhibited oxidative stress and depolarization of mitochondria evoked by 10 µM TBBPA, whereas these effects were only partially reduced in the 25 µM TBBPA treatment. Free radical scavengers prevented TBBPA-induced development of oxidative stress and improved CGC viability without having any effect on the rises in Ca^2+^ and drop in ∆Ψm. The co-administration of scavengers with NMDA and ryanodine receptor antagonists provided almost complete neuroprotection. These results indicate that Ca^2+^ imbalance and oxidative stress both mediate acute toxicity of TBBPA in CGC. At 10 µM TBBPA Ca^2+^ imbalance is a primary event, inducing oxidative stress, depolarization of mitochondria and cytotoxicity, whilst at a concentration of 25 µM TBBPA an additional Ca^2+^-independent portion of oxidative stress and cytotoxicity emerges.

**Electronic supplementary material:**

The online version of this article (doi:10.1007/s11064-016-2075-x) contains supplementary material, which is available to authorized users.

## Introduction

Tetrabromobisphenol A (2,2′,6,6′,-tetrabromo-4,4′-isopropylidine diphenol; TBBPA) is one of the main representatives of brominated flame retardants, substances that are additives to industrial products reducing their flammability. The mass production and use of TBBPA results in its leakage to the environment [1–3]. TBBPA has been detected in breast milk, in maternal serum samples [4, 5], and in the serum of workers having contacts with printed circuit boards [6]. The toxicity of TBBPA is controversial, in particular its possible neurotoxicity. It has been shown that TBBPA at low micromolar concentrations possesses cyto(neuro)toxic potential in vitro [7–11], whereas in vivo studies provide conflicting data as to its neurotoxic and neurodevelopmental effects [12–14].

TBBPA interferes with the activity of many proteins and signaling pathways which may mediate cyto(neuro)toxicity [7, 11, 15], and amongst the best-documented mechanisms of acute TBBPA neurotoxicity are impaired calcium homeostasis and oxidative stress [7, 9, 10]. Numerous studies have shown that TBBPA at low micromolar concentrations inhibits the activity of the sarcoplasmic/endoplasmic reticulum Ca^2+^ ATPase (SERCA) [8, 16] and voltage-gated calcium channels [15, 17]. TBBPA activates the release of Ca^2+^ from the intracellular ryanodine-sensitive stores via ryanodine receptors (RyR) [18, 19] and its influx to neurons via NMDA receptors (NMDAR) [10]. This results in increased intracellular Ca^2+^ concentration ([Ca^2+^]_i_) in cultured cells, including neurons, challenged with TBBPA [7, 10, 15, 18, 19].

TBBPA-evoked accumulation of ROS and of the products of free radical oxidation have been detected in vivo in liver and other organs of various species, and in vitro in cultured neuronal and non-neuronal cells [7–9, 15]. The mechanisms of these phenomena are unclear. In the present study we have focused only on the mechanism(s) of acute oxidative stress developing in primary neuronal cultures and other cell models challenged with TBBPA [7–9, 15]. Oxidative stress is an important element of excitotoxicity, which may result from the excessive activation of glutamate receptors, destabilization of cellular calcium homeostasis in neurons, and mitochondrial dysfunction [20–22]. Thus, there is a basis for a hypothesis that oxidative stress in neurons challenged with TBBPA may be secondary to TBBPA-evoked disorders of Ca^2+^ homeostasis [15, 23]. In addition to mitochondrial calcium overload which may lead to oxidative stress, the direct uncoupling of mitochondria by TBBPA resulting in enhanced Ca^2+^-independent ROS production in neurons has been postulated [31]. Our recent studies showed that simultaneous application of the antagonists of NMDAR and RyR completely prevents TBBPA-induced increases in [Ca^2+^]_i_ [10]. We, therefore, proposed that such a treatment could be instrumental in evaluating the role of TBBPA-evoked Ca^2+^ imbalance in inducing oxidative stress and cytotoxicity in neuronal cultures.

In the present study, primary cultures of rat CGC were used to determine the role of TBBPA-induced increases in [Ca^2+^]_i_ in the oxidative stress and cytotoxicity in neurons. The cultures were acutely challenged with TBBPA and we measured changes in [Ca^2+^]_i_, ROS production and selected indicators of oxidative stress, in the mitochondrial membrane potential (∆Ψm) and in the viability of neurons. The combination of NMDAR and RyR antagonists was applied to verify the hypothesis on the primary role of the TBBPA-induced increases in [Ca^2+^]_i_, while free radical scavengers were used to prevent oxidative stress and cyclosporin A was used to test the role of mitochondrial permeability transition pores (MPTP).

## Materials and Methods

### Materials

Ryanodine, (+)-5-methyl-10,11-dihydro-5H-dibenzo[a,d]·cyclohepten-5,10-imine hydrogen maleate (MK-801), butylated hydroxyanisole (BHA), reduced glutathione (GSH), ascorbic acid (vit. C), cyclosporin A (CsA), dimethyl sulphoxide (DMSO), fetal calf serum and other materials for cell culturing, were purchased from Sigma Chemical Poland (Poznan, Poland). TBBPA (99.8 % purity) was synthesized and delivered commercially by the Institute of Industrial Organic Chemistry, Analytical Department, in Warsaw, Poland. Bastadin 12 synthesized in the Laboratory of Natural Products Synthesis and Bioorganic Chemistry, Institute of Bioorganic Chemistry NCRS “Demokritos” in Athens, Greece, was kindly provided by Dr. Emmanuel N. Pitsinos. Fluorescent probes fluo-3/AM, rhodamine123 (Rh123) and 2′7′-dichlorodihydrofluorescein-diacetate (DCFH-DA) were produced by Molecular Probes Inc. (Paisley, UK). All other chemicals were of analytical grade.

### Animals

Primary CGC cultures were prepared from 7 day-old Wistar rats of the outbred stock CmD:(WI)WU. The animals were bred in the animal house of the Mossakowski Medical Research Centre, Polish Academy of Sciences, Warsaw under standard conditions i.e. fed and watered *ad libitum* and kept on a 12:12 h dark-light cycle, at room temperature with a constant humidity of approximately 60 %.

### Neuronal Cell Cultures

The cells were isolated and cultured according to a standard method [24] with slight modifications, exactly as has been described previously [9, 10, 19]. Briefly, the cells prepared from the cerebellar slices after tripsinization and trituration were suspended in basal Eagle medium supplemented with 10 % fetal calf serum, 25 mM KCl, 4 mM glutamine and antibiotics, then seeded onto 12-well plates coated with poly-L-lysine (NUNC) at a density of 2 × 10^6^ per well. The replication of non-neuronal cells was prevented by the application of 7.5 µM cytosine arabinofuranoside. The CGC cultures were used for experiments after 7 days in vitro.

### Fluorometric Measurements of Changes in [Ca^2+^]_i_, ROS Production and ∆Ψm

Changes in intracellular Ca^2+^ concentration ([Ca^2+^]_i_) in CGC were monitored using the fluorescent calcium-sensitive probe fluo-3. Its acetoxymethyl ester derivative, fluo-3 AM, easily penetrates plasma membranes, and inside the cells esterases cleave it to fluo-3, which becomes highly fluorescent after binding Ca^2+^ [24]. For the measurement of ROS production DCFH-DA was used. DCFH-DA is cleaved inside the cells to DCFH and further oxidized by ROS to the fluorescent product 2′7′-dichlorofluorescein (DCF) [25]. To evaluate changes in mitochondrial membrane potential (∆Ψm), rhodamine 1,2,3 (R123) was applied. Polarized mitochondria are known to accumulate R123 in a voltage-dependent way and bind this dye which results in quenching its fluorescence, whereas their depolarization leads to R123 release to the cytosol and restoration of its fluorescence [26].

**Fig. 1:**
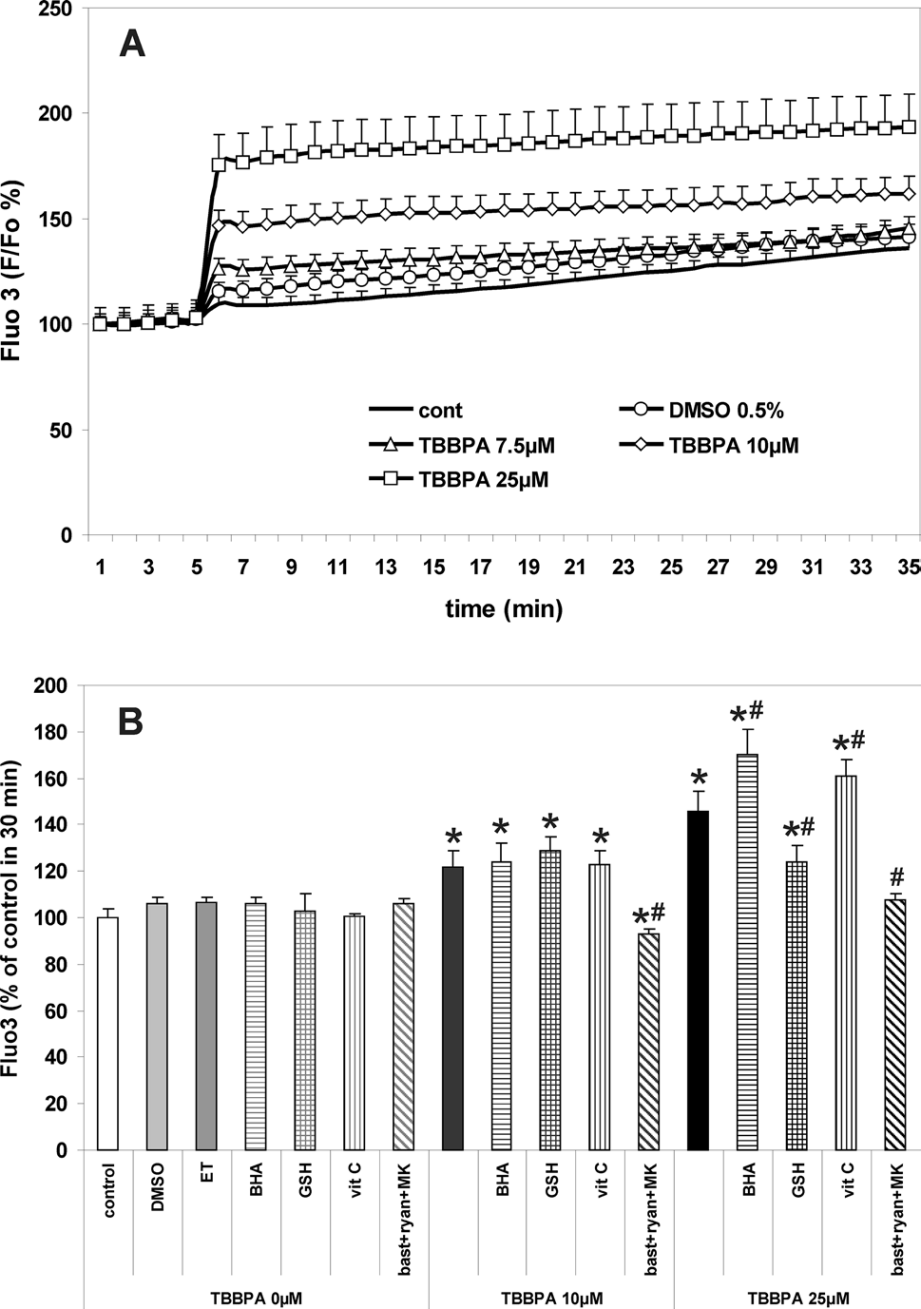
TBBPA-induced increases in intracellular Ca^2+^ concentration in primary CGC cultures. **a** The concentration-dependent effects of TBBPA versus vehicle (0.5 % DMSO) on the fluorescence of fluo-3. **b** Modulation of the effects of 10 and 25 µM TBBPA by the free radical scavengers 10 µM butylated hydroxyanisole (BHA) dissolved in 0.1 ‰ ethanol (ET), 1 mM reduced glutathione (GSH), 1 mM ascorbic acid (vit. C), and the combination of RyR and NMDAR antagonists 2.5 µM bastadin 12 (bast), 200 µM ryanodine (ryan) and 0.5 µM MK-801 (MK). Fluorescence of fluo-3 is expressed as percentage of the basal level (∆F/F_0_ %) (**a**), or percent of control at 30 min (**b**). The results are the mean values ± SD (n = 15). *Results significantly different from the control. ^#^Results significantly different from the corresponding group treated only with TBBPA (p < 0.05)

**Fig. 2:**
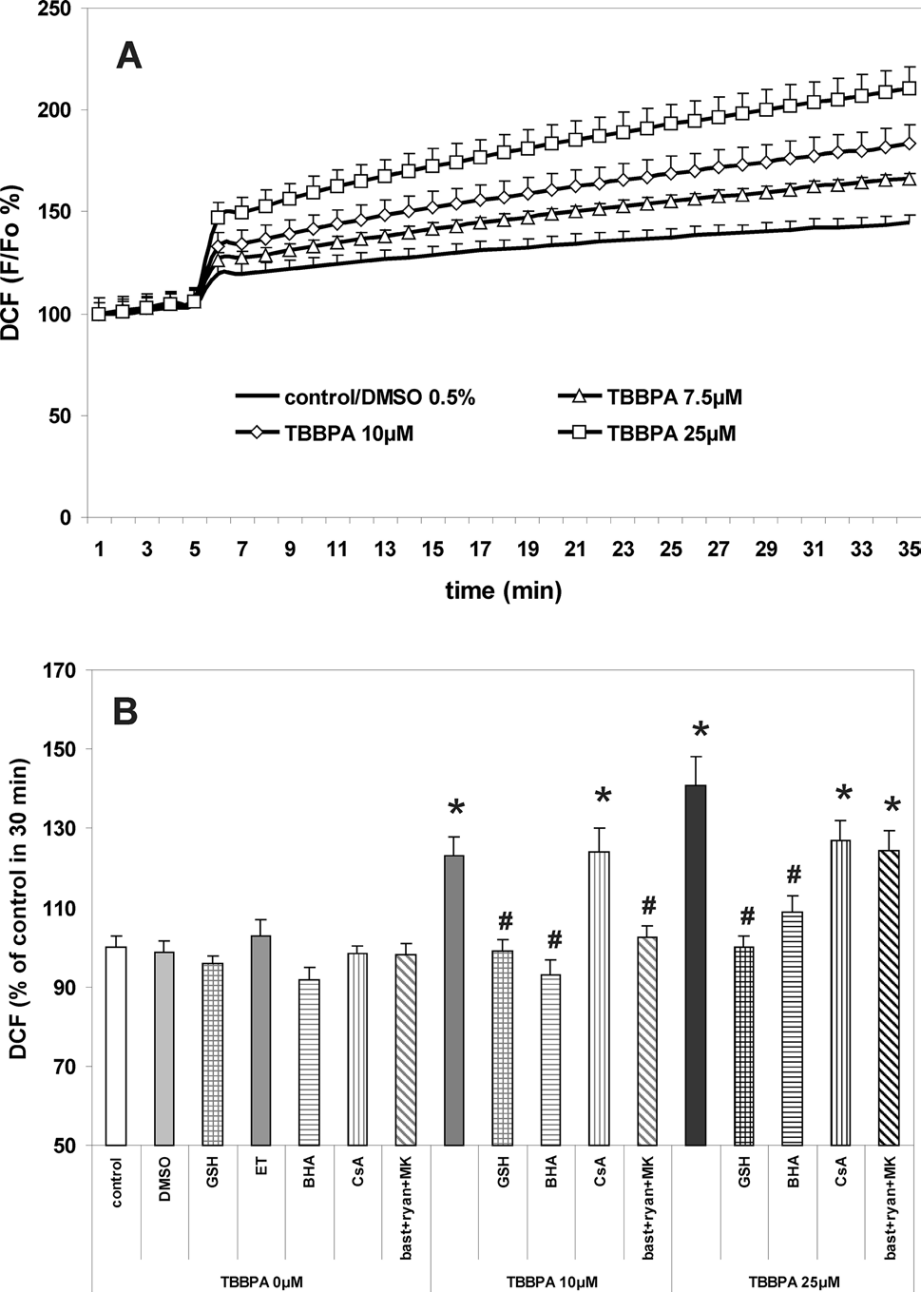
Increase in production of reactive oxygen species in primary cultures of CGC treated with TBBPA. **a** The concentration-dependent effects of TBBPA versus vehicle (0.5 % DMSO) on the fluorescence of DCF. **b** Modulation of the effects of 10 and 25 µM TBBPA by the free radical scavengers 1 mM reduced glutathione (GSH), 10 µM butylated hydroxyanisole (BHA) dissolved in 0.1 ‰ ethanol (ET), MPTP inhibitor 5 µM cyclosporin A (CsA), and the combination of RyR and NMDAR antagonists 2.5 µM bastadin 12 (bast), 200 µM ryanodine (ryan) and 0.5 µM MK-801 (MK). Fluorescence of DCF is expressed as percentage of the basal level (∆F/F_0_ %) (**a**), or percent of control at 30 min (**b**). The results are the mean values ± SD (n = 15). *Results significantly different from the control. ^#^Results significantly different from the corresponding group treated only with TBBPA (p < 0.05)

The procedure was essentially as has been described previously [9, 10, 27]. CGC cultures were incubated for 30 min at 37 °C in the original culture medium containing 4 μM fluo-3AM, 100 μM DCFH-DA or 10 μM R123. Then, the cultures were washed 3 times with Locke 5 buffer, containing 154 mM NaCl, 5 mM KCl, 2.3 mM CaCl_2_, 4 mM NaHCO_3_, 5 mM glucose and 5 mM HEPES (pH 7.4). The fluorescence of the cell-entrapped probes was measured using a microplate reader FLUOstar Omega (Ortenberg, Germany) set at 485 nm excitation and 538 nm emission wavelengths. Additional data concerning TBBPA-induced changes in fluo-3 and DCF fluorescence in CGC are provided in the supplementary material (Online Resource 2). After determining the baseline fluorescence of the cells incubated in Locke 5 buffer, the changes in fluorescence after the addition of the test compounds were recorded every 60 s. The results of fluorescence measurements are presented either as percent changes in fluorescence intensity relative to the basal level (F/F_0_ %) versus duration of measurement (Figs. 1a, 2a, 5a), or represent the level of fluorescence after 30 min of the experiment, in % of the control, i.e. the cells untreated with test substances or vehicles (bar graphs in Figs. 1b, 2b, 5b). The results of control experiments examining the effects of TBBPA and NMDAR/RyR antagonists on the fluorescence of cell-free solutions containing the fluorescent probes used in these studies are presented in the supplementary material (Online Resource 1).

### GSH Content in CGC

CGC cultures were incubated for 30 min at 37 °C in Locke 5 buffer in the presence of test compounds, then washed twice with PBS. Reduced glutathione (GSH) content was assayed using monochlorobimane, a thiol probe, using a Glutathione Assay Kit, Fluorimetric (Sigma-Aldrich, Saint Louis, Missouri, USA, Catalog Number CS1020). The manufacturers assay procedure was followed without modifications. Fluorescence was measured by a microplate reader set at 360 nm excitation and 485 nm emission wavelengths.

### Catalase Activity

CGC cultures were incubated for 30 min at 37 °C in Locke 5 buffer in the presence of test compounds, then washed twice with PBS. Cell lysates were prepared and catalase activity was assayed using a Catalase Assay Kit (Cayman Chemicals, Ann Arbor, MI, USA, Item No. 707002). The assay is based on the reaction of catalase with methanol in the presence of H_2_O_2_ and measurement of the produced formaldehyde with the chromogen Purpald [28].

### Acute TBBPA Cytotoxicity

The toxicity of TBBPA to CGC was assessed 24 h after 30 min exposure of the cultures, using propidium iodide staining [29], as described previously [9, 10]. Briefly, the growth medium in CGC cultures was replaced with Locke 25 buffer containing 134 mM NaCl, 25 mM KCl, 2.3 mM CaCl_2_, 4 mM NaHCO_3_, 5 mM HEPES (pH 7.4), 5 mM glucose, and, also, freshly prepared solutions of the test substances or vehicles, as required. After incubation at 37 °C for 30 min and two washes with Locke 25 buffer, the original growth medium was restored and the CGC were cultured for an additional 24 h. Then, the cells were fixed with 80 % methanol, stained with propidium iodide (0.5 µg/ml), and viable and dead neurons were counted using a fluorescence microscope Axiovert (Carl Zeiss AG, Germany), with the user blinded as to the experimental groups. The viability of the neurons as percentages of live cells in proportion to all cells was determined.

### Statistics

All experiments were repeated three times, and each time different preparations of CGCs from separate rat litters were used. Each treatment was replicated in five wells in each experiment. Because differences between these independent experiments were not statistically significant (one-way ANOVA, *P* < 0.05), the data were combined and analyzed as one set. All data are presented as the means ± SD with the same number of repetitions (n = 15). One-way ANOVA tests followed by Dunn’s method correction and the Wilcoxon rank test (Statistica software ver.10, StatSoft) were used to compare differences between experimental data points and the basal levels. *P* < 0.05 was considered significant for all tests.

## Results

### TBBPA-Induced Increases in [Ca^2+^]_i_ in CGC

The results presented in Fig. 1 show increases in fluo-3 fluorescence, which reflect corresponding rises in [Ca^2+^]_i_ resulting from the application of TBBPA to primary cultures of rat CGC. Persistent increases in [Ca^2+^]_i_ during 30-min incubation were dependent on TBBPA concentration (Fig. 1). The simultaneous application of 200 µM ryanodine with 2.5 µM bastadin 12 and 0.5 µM MK-801—which are antagonists of Ca^2+^ fluxes via leak RyRs channels and NMDARs, respectively—completely prevented increases in [Ca^2+^]_i_ induced by 10 µM TBBPA, and almost completely reduced the effect of 25 µM TBBPA (Fig. 1). These results presented here of measurements using a fluorescence plate reader are consistent with previously published data from real-time confocal microscopy [10], as well as with confocal microscopy measurements which we performed in the present study (see Online Resource 2).

Furthermore, Fig. 1 also shows that the increase in [Ca^2+^]_i_ induced by 25 µM TBBPA was significantly (*P* < 0.05) potentiated in the presence of the free radical scavengers 10 µM butylated hydroxyanisole (BHA) (55.5 % increase) or 1 mM vit. C (35.5 % increase). The addition of 1 mM GSH reduced calcium transients evoked by 25 µM TBBPA by 46.7 %. All these free radical scavengers had no effect on the [Ca^2+^]_i_ concentration in CGC untreated with TBBPA and on its increase evoked by 10 µM TBBPA (Fig. 1).

### TBBPA-Induced Increase in ROS Production in CGC

Oxidative stress in CGC challenged with TBBPA was assessed by measuring DCF fluorescence; its increase reflects ROS production. The results presented in Fig. 2 demonstrate the TBBPA concentration-dependent production of ROS during incubation of CGC with TBBPA. Figure 2 shows that ROS production in CGC induced by 10 or 25 µM TBBPA was differently modulated by the combination of NMDAR and RyR antagonists. This treatment, which completely prevented calcium transients induced by 10 µM TBBPA (Fig. 1), also totally inhibited the increase in ROS production. However, in the case of 25 µM TBBPA, almost the entire suppression of increases in the [Ca^2+^]_i_ level (Fig. 1) was accompanied by an insignificant tendency to reduce ROS production (Fig. 2). An additional experiment using confocal microscopy to measure DCF fluorescence in CGC challenged with TBBPA also exhibited an increase in the fluorescence (see Online Resource 2), this was dependent on TBBPA concentration and was completely prevented by NMDAR and RyR antagonists.

The free radical scavengers BHA and GSH completely prevented ROS production induced by 10 µM TBBPA, whereas with 25 µM TBBPA the production of ROS was totally inhibited in the presence of GSH, but less efficiently, and only by 78 %, when BHA was applied. Inhibitor of MPTP formation cyclosporin A (5 µM), had no significant effect on the production of ROS in CGC challenged with 10 and 25 µM TBBPA (Fig. 2).

### Effect of TBBPA on Antioxidant Potential of CGC

**Fig. 3:**
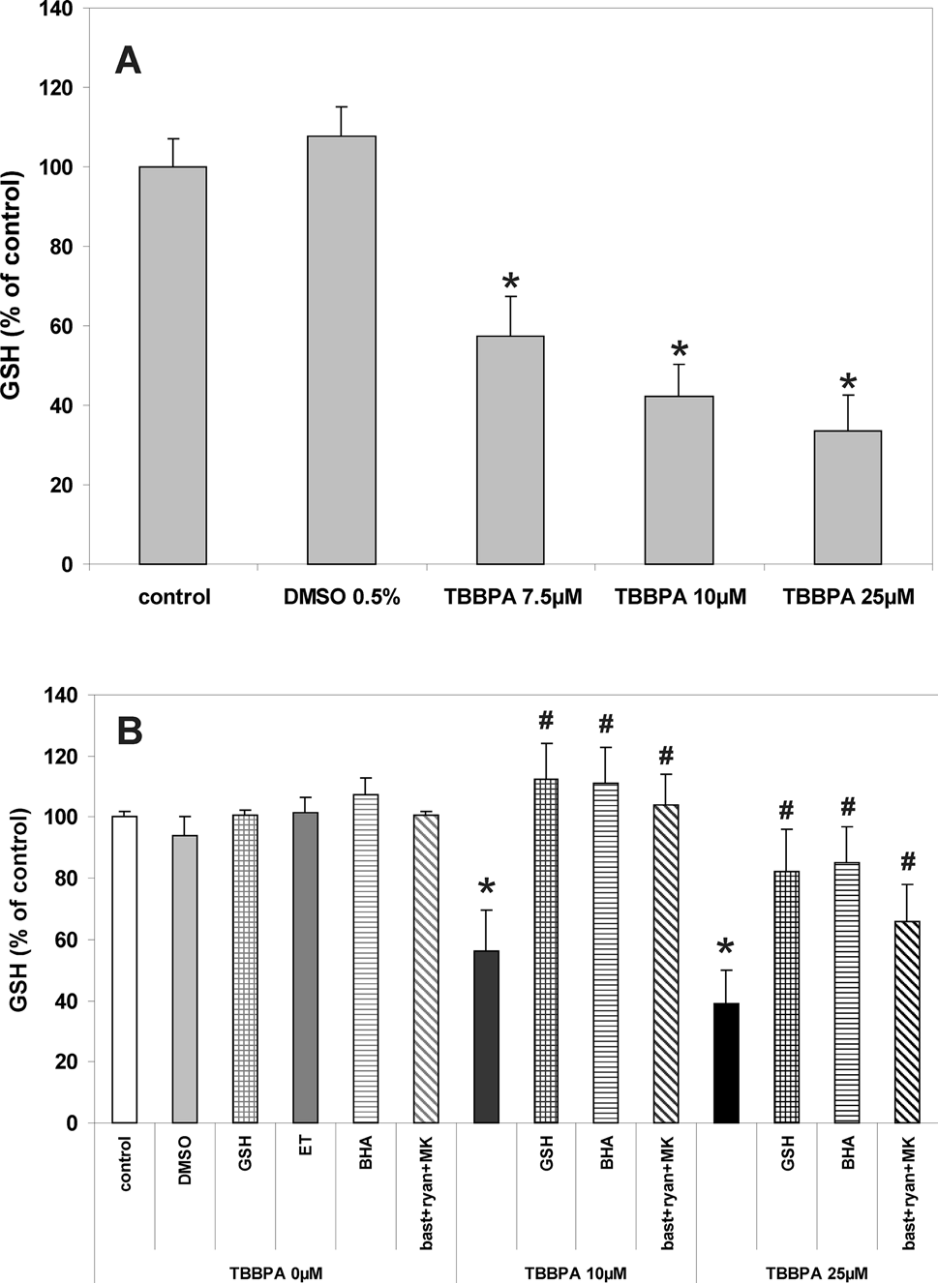
Decreases in the content of the reduced glutathione (GSH) in primary CGC cultures challenged for 30 min with TBBPA. **a** The effects of different concentrations of TBBPA and vehicle (0.5 % DMSO) on the GSH content. **b** Modulation of the effects of 10 and 25 µM TBBPA by the free radical scavengers 1 mM reduced glutathione (GSH) and 10 µM butylated hydroxyanisole (BHA) dissolved in 0.1 ‰ ethanol (ET), and by the combination of RyR and NMDAR antagonists 2.5 µM bastadin 12 (bast), 200 µM ryanodine (ryan) and 0.5 µM MK-801 (MK). GSH content in CGC is presented as percent of control (the untreated cultures). The results are the mean values ± SD (n = 15). *Results significantly different from the control. ^#^Results significantly different from the corresponding group treated only with TBBPA (p < 0.05)

In order to evaluate the endogenous antioxidant potential of CGC challenged with TBBPA, GSH content and catalase activity were assessed. As presented in Fig. 3, incubation of CGC with TBBPA results in a concentration-dependent decrease in the GSH content. The decrease in GSH level in CGC evoked by 10 µM TBBPA was completely prevented in the presence of NMDAR and RyR antagonists, and also in the presence of the exogenous free radical scavengers GSH and BHA (Fig. 3). Similar protective effects were exerted by other free radical scavengers, 1 mM N-acetylocysteine (NAC) and 1 mM vit. C (results not shown). A decrease in GSH content in the cultured neurons induced by 25 µM TBBPA was reduced only partially (by 44.3 %) by the mixture of bastadin 12, ryanodine and MK-801, and more efficiently, by 70.5 and 75.5 %, in the presence of GSH or BHA, respectively.

**Fig. 4:**
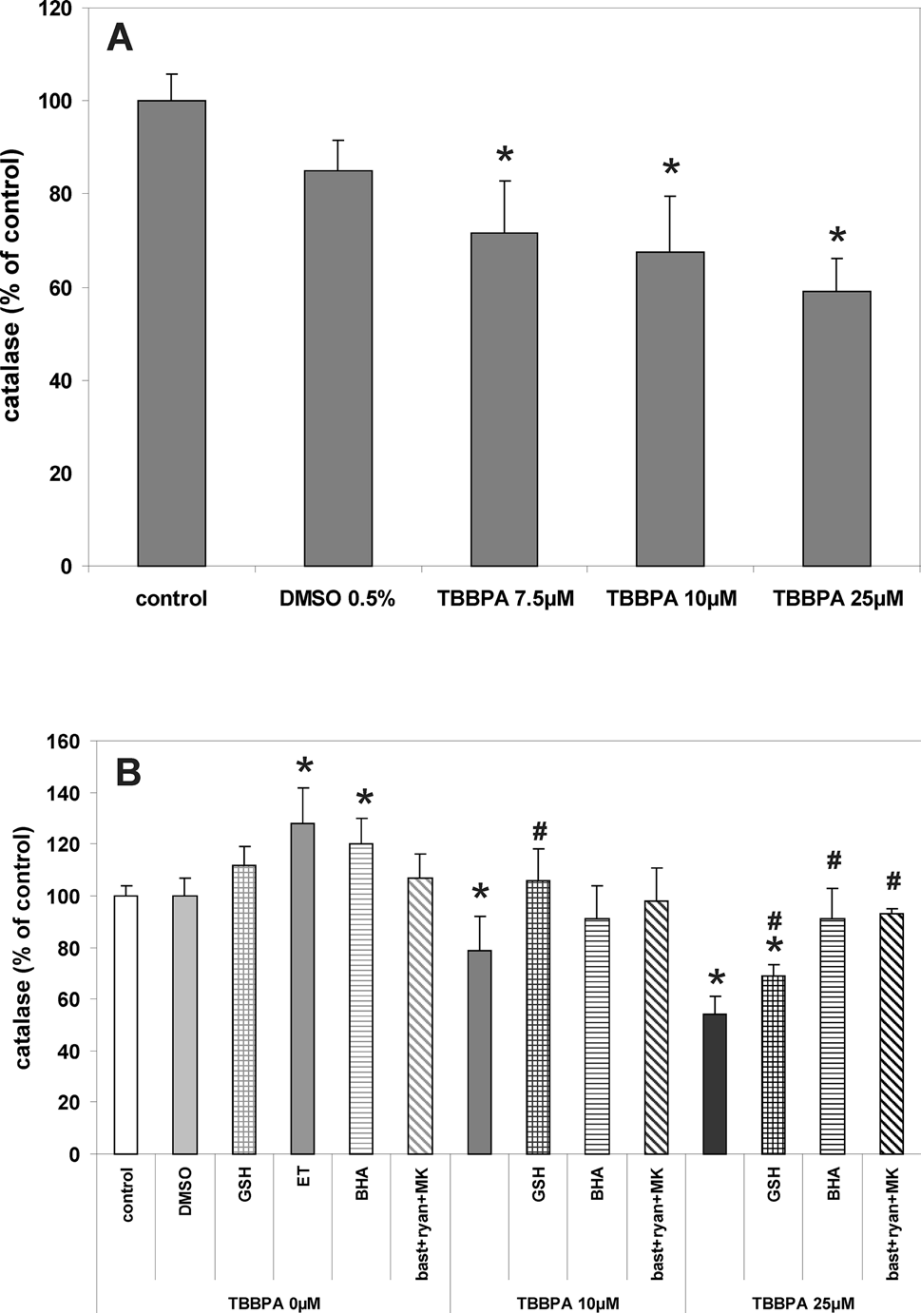
Decreases in catalase activity after 30-min incubation of CGC cultures with TBBPA. **a** The effects of different concentrations of TBBPA and vehicle (0.5 % DMSO) on the activity of catalase. **b** Modulation of the effects of 10 and 25 µM TBBPA by the free radical scavengers 1 mM reduced glutathione (GSH),10 µM butylated hydroxyanisole (BHA) dissolved in 0.1 ‰ ethanol (ET), and by the combination of RyR and NMDAR antagonists 2.5 µM bastadin 12 (bast), 200 µM ryanodine (ryan) and 0.5 µM MK-801 (MK). Catalase activity in CGC is presented as percent of control (the untreated cultures). The results are the mean values ± SD (n = 15). *Results significantly different from the control. ^#^Results significantly different from the corresponding group treated only with TBBPA (p < 0.05)

Incubation of CGC for 30 min with TBBPA (7.5, 10 and 25 µM) reduced catalase activity in a concentration-dependent manner, by 28, 32 and 41, respectively (Fig. 4). These effects induced by 10 µM TBBPA were completely reversed only by GSH, whereas a decrease in catalase activity induced by 25 µM TBBPA was significantly reduced by GSH, BHA and the combination of RyR and NMDAR antagonists (Fig. 3). Figure 4a also shows that there was a 20 % increase in catalase activity in control cells (no TBBPA treatment) after 30 min incubation in the presence of BHA. This is likely due to the effect of the ethanol (0.01 %) used as a vehicle for the BHA, since ethanol by itself increased catalase activity by 28 %.

### TBBPA-Induced Depolarization of Mitochondria

**Fig. 5:**
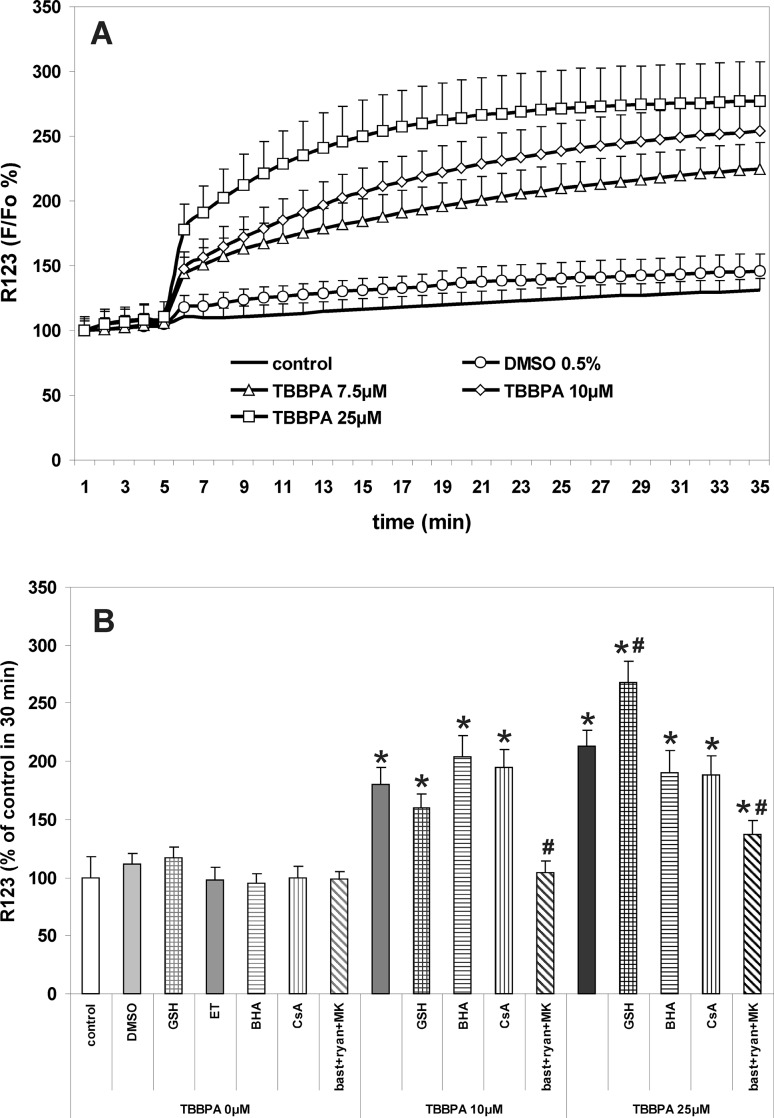
Depolarization of mitochondria in primary cultures of CGC incubated with TBBPA. **a** The concentration-dependent effects of TBBPA versus vehicle (0.5 % DMSO) on the fluorescence of rhodamine 123 (R123). **b** Modulation of the effects of 10 and 25 µM TBBPA by the free radical scavengers 1 mM reduced glutathione (GSH), 10 µM butylated hydroxyanisole (BHA) dissolved in 0.1 ‰ ethanol (ET), MPTP inhibitor 5 µM cyclosporin A (CsA), and the combination of RyR and NMDAR antagonists 2.5 µM bastadin 12 (bast), 200 µM ryanodine (ryan) and 0.5 µM MK-801. Fluorescence of R123 is expressed as percentage of the basal level (∆F/F_0_ %) (**a**), or percent of control at 30 min (**b**). The results are the mean values ± SD (n = 15). *Results significantly different from the control. ^#^Results significantly different from the corresponding group treated only with TBBPA (p < 0.05)

Changes in the mitochondrial membrane potential (∆Ψm) in CGC cultures treated with TBBPA were evaluated using the fluorescent probe rhodamine 123 (Rh123). As shown in Fig. 5a, the application of TBBPA resulted in a rapid concentration-dependent increase in the Rh123 fluorescence, which represents a decrease in ∆Ψm. The mixture of RyR and NMDAR antagonists completely prevented this effect in the 10 µM TBBPA treatment, whereas in the 25 µM TBBPA treatment depolarization of mitochondria was reduced by 67.3 % (Fig. 5). The free radical scavengers GSH and BHA had no statistically significant effects on depolarization of mitochondria in CGC treated with 10 µM TBBPA. In the 25 µM TBBPA treatment, a decrease in ∆Ψm was significantly potentiated in the presence of GSH, whereas BHA had no significant effect. The addition of 5 µM cyclosporin A, which is known to inhibit the formation of the mitochondrial permeability transition pores (MPTP), did not significantly prevent decreases in ∆Ψm induced by the 10 and 25 µM TBBPA treatments (Fig. 5).

### Reduction of Viability of CGC Challenged with TBBPA

**Fig. 6:**
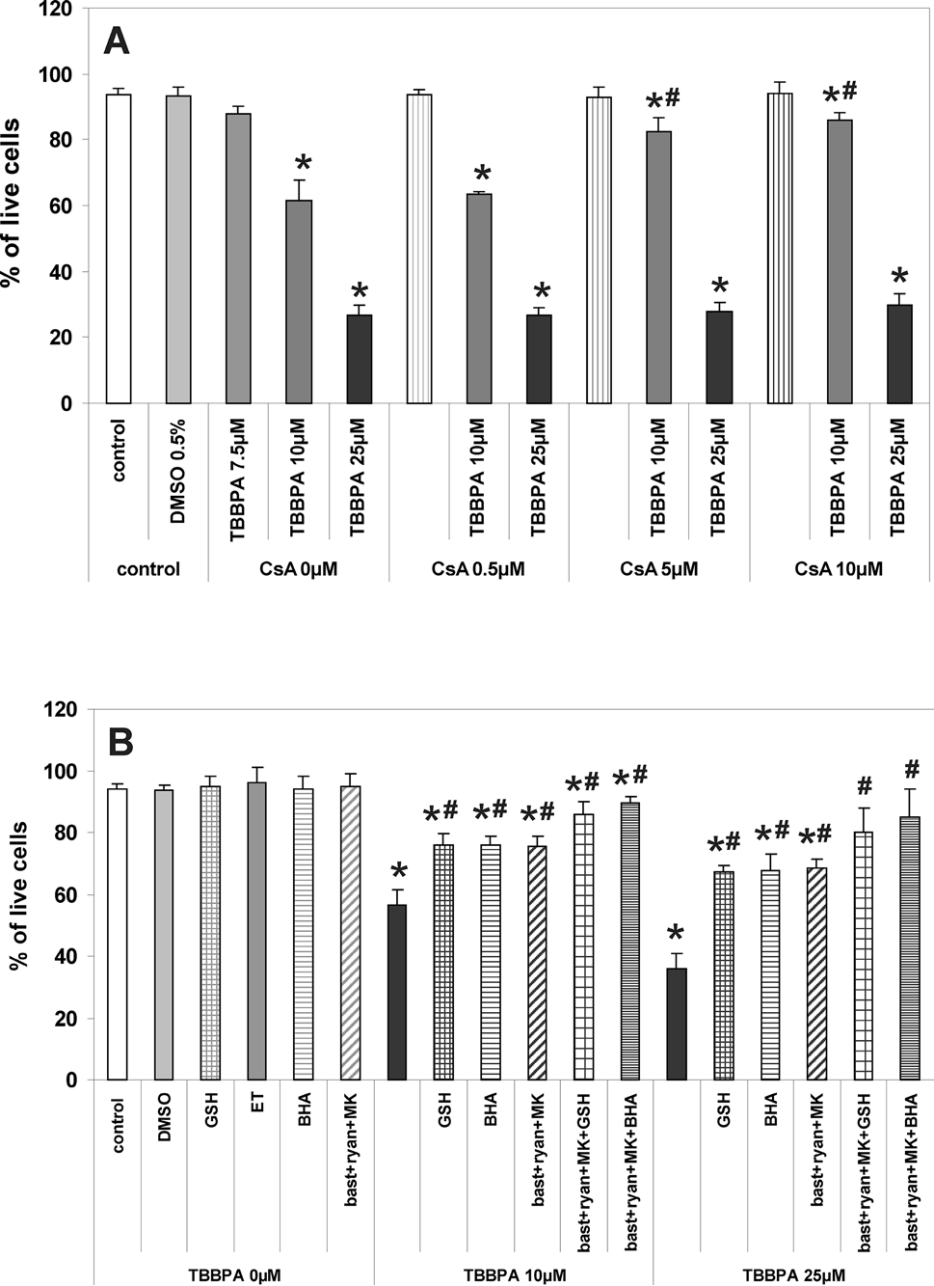
Reduction of viability of CGC cultures 24 h after 30-min exposure to TBBPA. **a** The concentration-dependent effects of TBBPA versus vehicle (0.5 % DMSO), and their modulation by MPTP inhibitor cyclosporin A (CsA). **b** Modulation of the effects of 10 and 25 µM TBBPA by the free radical scavengers 1 mM reduced glutathione (GSH), 10 µM butylated hydroxyanisole (BHA) dissolved in 0.1 ‰ ethanol (ET), the combination of RyR and NMDAR antagonists 2.5 µM bastadin 12 (bast), 200 µM ryanodine (ryan) and 0.5 µM MK-801, and by co-administration of the latter antagonists with GSH or BHA. Viability of CGC cultures is expressed as percentage of surviving cells. The results are the mean values ± SD (n = 15). *Results significantly different from the control. ^#^Results significantly different from the corresponding group treated only with TBBPA (p < 0.05)

Figure 6 shows that the acute treatment of CGC cultures with TBBPA results in a concentration-dependent decrease in the number of live neurons 24 h later. Antagonists of RyR and NMDAR, applied in combination, partially suppressed the decrease in neuronal viability induced by 10 and 25 µM TBBPA by 44.2 and 48.4 %, respectively (Fig. 6). A similar level of neuroprotection was observed in the presence of the free radical scavengers GSH or BHA. Co-application of RyR and NMDAR antagonists with GSH or BHA further increased neuronal viability in cultures treated with both concentrations of TBBPA, although complete neuroprotection was not achieved (Fig. 6). Figure 6 shows that 0.5 µM cyclosporin A had no neuroprotective effect, whereas 5 and 10 µM cyclosporin A strongly suppressed the decrease in neuronal viability; however, this neuroprotection was restricted to the 10 µM TBBPA treatment.

## Discussion

This study shows that in CGC TBBPA concentration-dependently increases [Ca^2+^]_i_, induces oxidative stress and depolarizes mitochondria, and that all these effects are completely (at 10 µM TBBPA) or very strongly (at 25 µM TBBPA) inhibited by a combination of NMDAR and RyR antagonists. These substances also provide significant, but incomplete protection of neuronal viability against TBBPA cytotoxicity. The administration of free radical scavengers does not inhibit TBBPA-induced calcium imbalance and mitochondrial depolarization, but the scavengers do reduce oxidative stress and provide significant neuroprotective effects. Co-administration of free radical scavengers with NMDAR and RyR antagonists almost completely prevents TBBPA cytotoxicity. The inhibitor of MPTP formation, cyclosporin A (5 µM), has no significant effect on TBBPA-induced ROS production and decrease in ∆Ψm, but it does reduce the cytotoxic effect of 10 µM TBBPA. These results demonstrate that both calcium imbalance and oxidative stress are involved in the mechanism(s) of TBBPA cytotoxicity in CGC. In the 10 µM TBBPA treatment they are indicative for a primary role of increases in [Ca^2+^]_i_ in triggering oxidative stress, depolarization of mitochondria and cytotoxicity; however, calcium-independent mechanisms emerge with a higher TBBPA concentration of 25 µM.

An in vitro model of the primary CGC cultures has been used in previous studies concerning TBBPA-evoked calcium imbalance and oxidative stress in neurons [7, 9, 10, 19] and, also, the effectiveness of blocking the TBBPA-induced calcium transients by the combination of NMDAR and RyR antagonists has been proven using CGC [10, 19]. The free radical scavengers GSH and butylated hydroxyanisole (BHA) have also been used at equivalent concentrations in mechanistic studies in cell cultures [27, 30]. Since different concentrations of cyclosporin A have been used in previous in vitro studies to inhibit MPTP formation [31, 32], here, for most experiments, a concentration of 5 µM cyclosporin A was selected based on its cytoprotective effect (Fig. 6). The fluorescent indicators fluo-3, DCF and R123—all measured with a plate reader—were used for the dynamic assessment of changes in CGC of [Ca^2+^]_i_, ROS production and ∆Ψm, respectively. Reports criticizing the low resolution and sensitivity of plate readers in measuring highly dynamic and transient changes in [Ca^2+^]_i_ with the fluorescent probes [33, 34] were considered; therefore, additional experiments were performed using real-time fluorescence microscopy to measure fluo-3 and DCF fluorescence in TBBPA-treated CGC. Both methods of measurement displayed very similar results (compare Figs. 1, 2 here and in Online Resource 2). In addition we have previously obtained comparable results between plate reading and real-time fluorescence microscopy when characterizing TBBPA-evoked increases in [Ca^2+^]_i_ in CGC, including the concentration-effect relation and pharmacological modulation [9, 10, 19]. Recent reports warn against the use of the DCF test to assess the stimulation of ROS production in cells by TBBPA [35–37]. It has been shown that TBBPA increases the fluorescence of DCFH-DA solutions in cell-free systems, which reflects the chemical interaction of TBBPA with the fluorescent indicator. This phenomenon was also observed in our own control experiments on cell-free DCFH-DA and DCF solutions (see Online Resource 1). However, in our opinion this purely chemical interaction does not alter the results and conclusions presented here, and we have provided a more detailed justification in the Online Resource 1. Furthermore, in order to assess more accurately oxidative stress in CGC challenged with TBBPA, in addition to the DCF test, we also examined selected indicators of endogenous antiradical potential, i.e. GSH content and catalase activity in CGC.

The present finding that increases in [Ca^2+^]_i_ in CGC are dependent on TBBPA concentration and may be totally prevented by co-application of the NMDAR antagonist 0.5 µM MK-801 with ligands of RyR, 200 µM ryanodine and 2.5 µM bastadin 12 (Fig. 1b), confirms our previously published results [10]. The results also support our claims that in CGC the mechanism of TBBPA-induced increases in [Ca^2+^]_i_ encompasses the release of Ca^2+^ from the ER stores via leak RyR and its influx to cells via NMDAR. A detailed discussion of these mechanisms has been presented previously [10, 19]. These present data justify the subsequent use of a combination of NMDAR and RyR antagonists in order to examine the causal relationships between the increase in [Ca^2+^]_i_ and ROS production in TBBPA-treated CGC. Furthermore, the results show that exogenously added free radical scavengers did not inhibit TBBPA-induced increases in [Ca^2+^]_i_ (Fig. 1) and suggest that TBBPA-induced oxidative stress is not implicated in the mechanism(s) of calcium imbalance in CGC challenged with TBBPA.

Oxidative stress is known to develop in CGC treated with TBBPA [7, 9]. In the present experiments, TBBPA concentration-dependently enhances ROS production (Fig. 2) and decreases GSH content and catalase activity in CGC (Figs. 3, 4). Both, GSH and catalase represent the antioxidant defense system of the cell, which encompasses non-enzymatic and enzymatic antioxidants [38]. We interpret a decrease in GSH content in TBBPA-treated CGC as an effect of excessive ROS production and subsequent GSH consumption in reducing hydrogen peroxide into H_2_O. GSH serves as a cofactor of several detoxifying enzymes [38], and in equilibrium with GSSH creates the major soluble antioxidant system in all cells. The redox imbalance has been suggested as a biomarker of several neurological diseases [39]. TBBPA-induced decrease in the GSH content or GSSG increase was observed in rat liver and in hepatocyte cultures [20] and in earthworm and aquatic species exposed to TBBPA [40]. Catalase, together with superoxide dismutases (SOD) and glutathione peroxidase (GSH-Px) are enzymatic antioxidants. SOD catalyze dismutation of the superoxide radical (O_2_^−^) into H_2_O_2_, whilst catalase and GSH-Px are responsible for converting this potentially toxic product into water [41]. Although the exact mechanism of the reduction of catalase activity in CGC acutely exposed to TBBPA is unknown, it is a secondary effect to increased ROS production, since the effect was reversed by exogenous free radical scavengers (Fig. 4). Because both the decrease in GSH content and reduced catalase activity are secondary to enhanced ROS production, these phenomena indirectly support our claims that TBBPA induces increased ROS production in CGC.

A key issue in the present study is the role of Ca^2+^ in the mechanism of the TBBPA-evoked oxidative stress. Acute challenging of human neutrophil granulocytes and CGC cultures with TBBPA was found to be accompanied by enhanced ROS formation, which was mediated by activation of several protein kinases and, in granulocytes, resulted from the increased activity of NADPH oxidase [7, 42]. It has been also demonstrated that TBBPA increases [Ca^2+^]_i_, and that incubation of cells in Ca^2+^-free medium reduces ROS production, suggesting the role of calcium in inducing oxidative stress, although MK-801 failed to significantly inhibit ROS production in CGC treated with 10 µM TBBPA [7, 15, 42]. The results of our present experiments revealed that the combination of RyR and NMDAR antagonists, which prevented calcium transients in CGC induced by 10 µM TBBPA (Fig. 1), also completely inhibits all the indices of oxidative stress (Figs. 2, 3, 4). This is consistent with the hypothesis of the primary role of increases in [Ca^2+^]_i_ in triggering TBBPA-evoked oxidative stress in CGC. Also, under these conditions, the free radical scavengers GSH and BHA prevented accumulation of ROS and a decrease in the antiradical potential of the cells. In contrast, with 25 µM TBBPA, despite the almost complete inhibition of the increase in [Ca^2+^]_i_ by the combination of NMDAR and RyR antagonists, these compounds only partially reversed an increase in the production of ROS and a decrease in GSH level (Figs. 2, 3). Also the protective effects of free radical scavengers against oxidative stress induced by 25 µM TBBPA were mostly limited and incomplete. These results suggest that, in addition to the Ca^2+^-mediated mechanism, at 25 µM TBBPA Ca^2+^-independent processes potentiating oxidative stress emerge. It is possible, that a portion of the Ca^2+^-independent increase of DCF fluorescence induced by 25 µM TBBPA can be attributed to the direct chemical interactions of TBBPA with the cells, since this has been found to exhibit free radical-like properties [37].

An increase in [Ca^2+^]_i_ in cells can lead to Ca^2+^accumulation in mitochondria and, subsequently, mitochondrial calcium overload results in a decrease in ∆Ψm, induction of MPTP and enhanced ROS production in mitochondria [43, 44]. In addition, high concentrations of TBBPA may directly uncouple oxidative phosphorylation in mitochondria and induce lipid peroxidation, as has been suggested in relation to hepatocytes [20]. Our present results show that TBBPA depolarizes mitochondria in a concentration-dependent manner (Fig. 5), and that, at a concentration of 10 µM TBBPA, depolarization is prevented in the presence of a combination of NMDAR and RyR antagonist that preclude TBBPA-evoked increases in [Ca^2+^]_i_. These results indicate that an increase in [Ca^2+^]_i_ is a trigger for mitochondrial depolarization. The co-existence of TBBPA-induced increases in [Ca^2+^]_i_ and ROS production, with depolarization of the mitochondria and cytochrome c release, were demonstrated previously using the SH-SY5Y human neuroblastoma cell line [8]; however, to our knowledge, the present study is the first to show the direct causal relationship between calcium transients and mitochondrial depolarization in neurons treated with low µM concentrations of TBBPA. In addition, our results show that a decrease in ∆Ψm induced by 25 µM TBBPA may be only incompletely reversed by inhibitors of NMDAR and RyR, suggesting partial involvement of Ca^2+^-unrelated mechanisms. Most likely 25 µM TBBPA directly depolarizes the mitochondrial membranes [20]. Indeed, TBBPA at µM concentrations was found to change the properties of phospholipid membranes [16], and the depolarizing effect of TBBPA on synaptosomal plasma membranes has been reported [45]. We have also demonstrated that the application of the free radical scavengers GSH and BHA, which dramatically inhibit ROS levels in CGC challenged with TBBPA, has no effect on the TBBPA-induced drop in ∆Ψm (Fig. 5), which is consistent with the explanation that mitochondrial calcium overload, not oxidative stress, triggers the drop in ∆Ψm in TBBPA-treated CGC. Also cyclosporin A applied at a neuroprotective concentration of 5 µM (Fig. 6) had no effect on TBBPA-induced mitochondrial depolarization (Fig. 5) and ROS formation (Fig. 2), which does not support the role of MPTP in TBBPA-induced mitochondrial dysfunction and oxidative stress in CGC. However the efficacy of cyclosporin A as an inhibitor of MPTP formation in the mitochondria of neurons is controversial [46, 47]. Previously it has been shown that 2 µM cyclosporin A effectively prevents the induction of MPTP in astrocytic mitochondria, but not in primary CGC in culture [32]; thus, the question of whether induction of MPTP plays a role in TBBPA-induced ROS production in CGC remains unresolved and requires further studies.

In agreement with previously published data [7, 10, 11, 17] we showed TBBPA cytotoxicity to be concentration dependent and suppressed significantly, but incompletely by a combination of NMDAR and RyR antagonists (Fig. 6). Thus, in addition to Ca^2+^-mediated cytotoxicity in CGC challenged with TBBPA, there are also other additional mechanisms, presumably related to oxidative stress [10]. Indeed, our results confirmed this and show that the free radical scavengers GSH (1 µM) and BHA (10 µM) have a strong neuroprotective potential. Furthermore, Reistad and coauthors [7] have shown that vitamin E also provides partial protection against TBBPA cytotoxicity in CGC. Of great importance here, we have shown that free radical scavengers co-applied with a combination of NMDAR and RyR antagonists collectively provide almost complete neuroprotection (Fig. 6). These data indicate that calcium-dependent and calcium-independent mechanisms can trigger oxidative stress and that both are involved in TBBPA cytotoxicity. When CGC were challenged with 10 µM TBBPA the application of cyclosporin A at a concentration of both 5 and 10 µM did significantly enhance CGC survival; however, this was not the case when the CGC were challenged with 25 µM TBBPA. Because the application of cyclosporin A at a concentration of 5 µM failed to reduce the TBBPA-induced drop in ∆Ψm and ROS production in CGC, we do not link these neuroprotective effects with inhibition of MPEP formation. Cyclosporin A also blocks the activation of calcineurin [48], and it is known that calcineurin inhibitors provide protection to neurons [49]. It should be emphasized that the concentrations of 10 and 25 µM TBBPA used in these experiments—selected on the basis of our own publications [9, 10]—are several orders of magnitude higher than those found in human body fluids [15]. Thus, although there are views that the cumulative toxic effect of TBBPA and neurotoxins with a similar mechanism of action, such as polychlorinated biphenyls and organic mercury compounds, can be much stronger than the effects of toxins examined individually [23], however, the results of this work, applicable only for TBBPA, have little relevance to human risk assessment.

In summary, the results of this study demonstrate that in CGC calcium imbalance and oxidative stress both mediate acute cytotoxicity of TBBPA, and that a causal relationship between these mechanisms depends on the concentration of TBBPA. At a concentration of 10 µM TBBPA, an increase in [Ca^2+^]_i_—resulting from influx of Ca^2+^ via NMDAR and its efflux from the ER stores via dysfunctional RyR—is a primary event triggering oxidative stress, depolarization of mitochondria and reduction of the neuronal viability. Simultaneous occurrence of neurotoxicity, impaired calcium homeostasis and oxidative stress in neurons exposed to TBBPA has been described previously [7], and the primary role of disorders of calcium homeostasis has been suggested (for review see [23]). Our present work, which uses selective pharmacological tools that completely abolish the TBBPA induced calcium transients in CGC, for the first time directly demonstrates these relationships. At a concentration of 25 µM TBBPA additional calcium-independent mechanisms of induction of oxidative stress and cytotoxicity emerge. The role of mitochondrial dysfunction in TBBPA-induced oxidative stress and cytotoxicity in CGC should be considered; however, the involvement of MPTP remains unclear. Although the results obtained here using an in vitro neuronal culture model are not relevant as regards the toxic effect of TBBPA on the brain of living organisms, nevertheless, they reveal interesting interactions between different mechanisms of cytotoxicity in neurons triggered by TBBPA.

## Electronic supplementary material

Below is the link to the electronic supplementary material.

Supplementary material 1 (PDF 300 KB)

Supplementary material 2 (PDF 436 KB)
