# Supplementary material for: The Role of Ca2+ Imbalance in the Induction of Acute Oxidative Stress and Cytotoxicity in Cultured Rat Cerebellar Granule Cells Challenged with Tetrabromobisphenol A
Source: Neurochem Res. 2016 Oct 7;42(3):777–87. doi: 10.1007/s11064-016-2075-x (PMC5357503; doi:10.1007/s11064-016-2075-x)
Supplement: Supplementary file 1 — Supplementary material 1 (PDF 300 KB) [file 11064_2016_2075_MOESM1_ESM.pdf]

Online Resource 1

Supplementary material to the Neurochemical Research article "The role of Ca^{2+} imbalance in the induction of acute oxidative stress and cytotoxicity in cultured rat cerebellar granule cells challenged with tetrabromobisphenol A"

Authors: Elzbieta Zieminska, Jacek Lenart, Dominik Diamandakis, Jerzy W. Lazarewicz

Affiliation: Department of Neurochemistry, Mossakowski Medical Research Centre, Polish Academy of Sciences, Warsaw, Poland.

E-mail of the corresponding author: jerzyl@imdik.pan.pl

Interference of TBBPA, NMDAR and RyR antagonists with fluorescence of Ca^{2+}-, ROS- and ΔΨm-sensitive probes in cell-free solutions

The main article deals with the role of intracellular calcium transients evoked by TBBPA in the induction of oxidative stress and mitochondrial dysfunction in primary cultures of CGC. To evaluate the effects of TBBPA on $[Ca^{2+}]_i$, ROS production and mitochondrial membrane potential (ΔΨm), the following fluorescent probes were used: fluo-3 AM, DCFH-DA and rhodamine 123, respectively. A particularly sensitive issue is the use in these studies of DCFH-DA.

The acetylated derivative of fluorescein, DCFH-DA, readily penetrates cell membranes and is subject to deacetylation once inside the cells, then, in the presence of free radicals, it is converted to the fluorescent product DCF. The measurement of DCF fluorescence is widely used to evaluate the production of ROS in cells [1-3], including oxidative stress induced by TBBPA [4-6]. However, recently it has been shown that TBBPA induces fluorescence in cell-free DCFH-DA solutions, and on this basis it has been concluded that the DCF assay cannot be used to evaluate cellular ROS production in TBBPA studies [7-

9]. In addition, the presence of bromine atoms in the structure of TBBPA means that it acts like a free radical molecule and is susceptible to reductive debromination [9]. Therefore, the interaction of TBBPA with other fluorescent probes used in this study cannot be excluded. Moreover bastadin 12, one of the pharmacological tools used in this study that modulates the activity of RyR, is a brominated tyrosine derivative which could also react with the fluorescent probes.

The primary aim of these control experiments was to assess the direct interaction of TBBPA with DCFH-DA with regards to the increase in DCF fluorescence observed in the CGC challenged with TBBPA. The experiments have also been extended to study the effects other key pharmacological substances used in the main article may have on the fluorescence of DCFH-DA and other fluorescent probes. Therefore, using cell-free solutions we evaluated the effects of TBBPA, MK-801, bastadin 12 and ryanodine on the fluorescence of DCFH-DA, DCF, fluo-3 AM, fluo-3, and rhodamine 123. The effects of TBBPA on the DCF test will be discussed in relation to the experiments on CGC cultures, described in the main article.

In all the experiments TBBPA was applied at the same concentration that was used in the main article, 10 μM and 25 μM. In the initial experiment a 100 μM solution of DCFH-DA and DCF was used, which is the loading concentration of the DCF assay in CGC (see the article). In other experiments (Tables 2-4) the probes were used at arbitrarily selected concentration of 1 μM. In the cell-free experiments DCFH-DA, DCF, fluo-3 AM, fluo-3 and rhodamine 123 were dissolved in Locke 5 buffer and dispensed into 96-well plates and after determining the baseline fluorescence, TBBPA or other test substances were applied. The fluorescence was measured after 1 and 30 min using a microplate reader FLUOstar Omega set at 485 nm excitation and 538 nm emission wavelengths. The results of measurements after 30 min were normalized to the auto-fluorescence of the test substances and expressed as a percentage of the fluorescence of a control sample.

Table 1. Effects of TBBPA and NMDAR/RyR antagonists on the fluorescence of 100 μM DCFH-DA solution in the cell-free system

Treatment	fluorescence at 30 min (% of control)
Control	100
DMSO 0.5 %	98
TBBPA 10 μM	110
TBBPA 25 μM	110
MK-801+ryan+ bast	103

The fluorescence was measured in cell-free Locke 5 buffer containing 100 μM DCFH-DA in the absence (control, DMSO) or presence of 10 or 25 μM TBBPA or a combination of 0.5 μM MK-801, 200 μM ryanodine and 2.5 μM bastadin 12. The results are from one experiment and means are calculated from 3 replicates per treatment.

The results shown in Table 1 demonstrate that 0.5 % DMSO had no effect on the fluorescence of DCFH-DA solution, whereas the application of TBBPA resulted in an increase in the fluorescence to 110%. Under these conditions TBBPA had no effect on the fluorescence of 100 μM DCF (results not shown). There was little effect on the fluorescence using the combination of NMDAR and RyR antagonists, 0.5 μM MK-801, 200 μM ryanodine and 2.5 μM bastadin 12.

In subsequent experiments DCFH-DA and other fluorescent probes were used at a concentration of 1 μM. The results presented in Table 2 demonstrate that TBBPA at both concentrations, as well as the other brominated substance, bastadin 12 increased the fluorescence of the 1 μM cell-free solution of DCFH-DA by 32-40% and of DCF by 8–14 %. The vehicle, DMSO, and the other test substances did not interfere with DCFH-DA and DCF fluorescence.

Table 2. Effects of TBBPA and NMDAR/RyR antagonists on the fluorescence of 1 μM DCFH-DA and DCF solutions in the cell-free system

Treatment	fluorescence at 30 min (% of control)	
	DCFH-DA	DCF
Control	100	100
DMSO 0.5 %	97	104
TBBPA 10 μM	132	108
TBBPA 25 μM	134	108
MK-801	94	104
Ryanodine	98	104
Bastadin 12	140	114

The fluorescence was measured in cell-free Locke 5 buffer containing 1 μM DCFH-DA or 1 μM DCF in the absence (control, DMSO) or presence of 10 or 25 μM TBBPA, 0.5 μM MK-801, 200 μM ryanodine and 2.5 μM bastadin 12. The results are from one experiment and means are calculated from 3 replicates per treatment.

Table 3. Effects of TBBPA and NMDAR/RyR antagonists on the fluorescence of 1 μM fluo-3 AM and fluo-3 solutions in the cell-free system

Treatment	fluorescence at 30 min (% of control)	
	fluo-3 AM	fluo-3
Control	100	100
DMSO 0.5 %	98	97
TBBPA 10 μM	107	106
TBBPA 25 μM	106	103
MK-801	100	98
Ryanodine	98	95
Bastadin 12	105	111

Fluorescence was measured in cell-free Locke 5 buffer containing 1 μM fluo-3 AM or fluo-3 in the absence (control, DMSO) or presence of 10 or 25 μM TBBPA and 0.5 μM MK-801, 200 μM ryanodine and 2.5 μM bastadin 12. The results are from one experiment and means are calculated from 3 replicates per treatment.

In the next experiment (Table 3) the effects of the test substances on the fluorescence of cell-free solutions containing 1 μM fluo-3 AM and fluo-3 were measured. Table 3 shows

that TBBPA very slightly increased the fluorescence by 3 – 7%, whereas bastadin 12 enhanced fluo-3 fluorescence by 11%.

Table 4 shows the effects of the test substances on the fluorescence of rhodamine 123 in cell-free solutions. TBBPA (10 and 25 μM) concentration-dependently reduced the fluorescence by 23 and 31 %, respectively. The other brominated substance, bastadin 12, only slightly decreased the fluorescence by 9%.

Table 4. Effects of TBBPA and NMDAR/RyR antagonists on the fluorescence of 1 μM rhodamine 123 solution in the cell-free system

Treatment	fluorescence at 30 min (% of control)
Control	100
DMSO 0.5 %	98
TBBPA 10 μM	77
TBBPA 25 μM	69
MK-801	98
Ryanodine	99
Bastadin 12	91

The fluorescence was measured in cell-free Locke 5 buffer containing 1 μM rhodamine 123, in the absence (control, DMSO) or presence of 10 or 25 μM TBBPA, 0.5 μM MK-801, 200 μM ryanodine and 2.5 μM bastadin 12. The results are from one experiment and means are calculated from 3 replicates per treatment.

These data confirm the recent results of other authors [7-9] that TBBPA increases the fluorescence of DCFH-DA solutions in cell-free systems. They also demonstrated that there is a less pronounced TBBPA-evoked increase in the fluorescence of DCF solution, and that the other brominated compound bastadin 12 also potentiates the fluorescence of DCFH-DA solution. In addition, TBBPA and bastadin 12, which only weakly enhanced the fluorescence of fluo-3 AM and fluo-3 solutions, reduced the fluorescence of rhodamine 123. The question is whether the data obtained in CGC cultures using the DCF test are

representative of a biological response, or whether they are artifacts of the purely chemical interaction between the test substances and the fluorescent probes. Other authors have warned that the increased DCF fluorescence observed with TBBPA in the presence of cells cannot be attributed to cellular ROS [7], and that the DCF test is not suitable for evaluating TBBPA-induced oxidative stress in cells [9]. We argue that the ROS using DCF test is still useful, provided the results are treated with caution. These results of biological experiments should be additionally confirmed using pharmacological tools that do not interfere directly with oxidative stress. Moreover assessing the level of oxidative stress with alternative methods should provide supporting data. In our study, these conditions have been met.

In our opinion simple control experiments using cell-free DCFH-DA or DCF solutions do not provide information as to the extent the results of experiments on cells may be distorted. Someone who recognizes that a TBBPA – DCFH-DA interaction can affect the results of DCF tests in cellular models should use concentrations of DCFH-DA or DCF solutions that correspond to intracellular concentrations of these indicators. It is because the relative increases in fluorescence (in percent of control) of DCFH-DA solutions induced by TBBPA were shown to be inversely proportional to the concentration of the indicator [7, 9]. However, the actual concentration of DCFH-DA inside the cells after CGC loading is unknown. In our control experiments in cell-free systems in the presence of TBBPA we observed 10% or 32 – 34 % increase in fluorescence using arbitrarily selected concentrations of DCFH-DA of 100 and 1 μM, respectively. However these results cannot be directly related to the situation inside the cells because, as has been mentioned above, the actual concentration of DCFH-DA/DCF within the cells is not known.

The results of our experiments described in the main paper and in the supplementary material show that there are some important incompatibilities between DCF

and rhodamine 123 tests in the cell-free system and those from experiments using the CGC cultures. Firstly, NMDAR and RyR antagonists that in CGC cultures inhibit increases in $[Ca^{2+}]_i$ induced by 10 μM TBBPA, but have no known antiradical potential, also prevented TBBPA-evoked increases in DCF fluorescence in the cells (the main article, Figs 1 and 2). One should also consider, that the combination of these antagonists also including bastadin 12, does not interfere with the fluorescence of DCF in the control, TBBPA-untreated CGC (Fig. 2). In turn, in the cell-free system, two of these antagonists, MK-801 and ryanodine, also had no effect on the fluorescence of the 1 μM solution of DCFH-DA, whereas bastadin 12 greatly enhanced the fluorescence. The other example of inconsistent results concerns the effect of TBBPA on rhodamine 123 fluorescence. In the cell-free solutions the fluorescence of rhodamine 123 was decreased by 23 – 31% in the presence of TBBPA (Online Resource 1, Table 4), whereas in experiments on CGC cells TBBPA strongly increased fluorescence of this probe, indicating mitochondrial depolarization (the main article, Fig. 5). Thus, several results from our biological experiments on CGC, particularly those using pharmacological tools, are not consistent with data from the cell-free system and, collectively, they do not support the concerns arising from the control experiments in cell-free systems. We propose the following interpretation as to the reasons for these discrepancies. In the case of TBBPA and bastadin 12, the results obtained from experiments in cell-free systems are not applicable to the situation actually in the cells. TBBPA and bastadin 12 are highly hydrophilic and in the cells they bind to the membranes, whereas the products of DCFH-DA deacylation are dissolved in the cytosol. Clearly, then, these substances are located in different cell compartments and the possibility of their direct interaction is rather weak. Thus, the results obtained from the experiments in cell-free solutions alone do not explain the extent to which a direct TBBPA-DCFH-DA interaction interferes with the results of the DCF test in cells treated with TBBPA. In our opinion, there is no evidence that direct interactions of TBBPA

with the fluorescent indicators significantly alters the pattern of the impact of TBBPA on ROS production, the level of $[Ca^{2+}]_i$ and $\Delta\Psi m$ in CGC observed in our study.

The presentation of increased production of ROS in the DCF test solely, without support of other indices, would be incomplete proof for TBBPA-induced oxidative stress in CGC. Our results using the DCF test are consistent with results where alternative methods have been used to evaluate oxidative stress. The results described in the main article (Figs 3 and 4) show that in addition to an increase in DCF fluorescence, TBBPA induces a concentration-dependent decrease in GSH level and catalase activity in CGC, and that these effects of 10 μM TBBPA are eliminated by the NMDAR and RyR antagonists. Based on literature data and our own results we consider a drop in GSH level and decreased catalase activity to be secondary to increased production of ROS. Our results strongly suggest that an increase in DCF fluorescence in CGC treated with 10 μM TBBPA is not an artefact, but reflects the increased production of ROS, which is an element of TBBPA-induced, Ca^{2+}-mediated oxidative stress in neurons.

References

[1] Wang H, Joseph JA (1999) Quantifying cellular oxidative stress by dichlorofluorescein assay using microplate reader. Free Radic Biol Med 27:612-616.

[2] Aam BB, Fonnum F (2006) (+/-)-2-Chloropropionic acid elevates reactive oxygen species formation in human neutrophil granulocytes. Toxicology 228:124-134.

[3] Kalyanaraman B, Darley-Usmar V, Davies KJ, Dennery PA, Forman HJ, Grisham MB, Mann GE, Moore K, Roberts LJ 2nd, Ischiropoulos H (2012) Measuring reactive oxygen and nitrogen species with fluorescent probes: challenges and limitations. Free Radic Biol Med 52:1-6.

[4] Reistad T, Mariussen E, Ring A, Fonnum F (2007) In vitro toxicity of tetrabromobisphenol-A on cerebellar granule cells: cell death, free radical formation, calcium influx and extracellular glutamate. Toxicol Sci 96:268-278.

[5] Ziemińska E, Stafiej A, Toczyłowska B, Lazarewicz JW (2012) Synergistic neurotoxicity of oxygen-glucose deprivation and tetrabromobisphenol A in vitro: role of oxidative stress. Pharmacol Rep 64:1166-1178.

[6] Hendriks HS, van Kleef RG, van den Berg M, Westerink RH (2012) Multiple novel modes of action involved in the in vitro neurotoxic effects of tetrabromobisphenol-A. Toxicol Sci 128:235-246.

[7] Tetz LM, Kamau PW, Cheng AA, Meeker JD, Loch-Caruso R (2013) Troubleshooting the dichlorofluorescein assay to avoid artifacts in measurement of toxicant-stimulated cellular production of reactive oxidant species. J Pharmacol Toxicol Methods 67:56-60.

[8] Szychowski KA, Wójtowicz AK (2016) TBBPA causes neurotoxic and the apoptotic responses in cultured mouse hippocampal neurons in vitro. Pharmacol Rep 68:20-26.

[9] Szychowski KA, Rybczyńska-Tkaczyk K, Leja ML, Wójtowicz AK, Gmiński J (2016) Tetrabromobisphenol A (TBBPA)-stimulated reactive oxygen species (ROS) production in cell-free model using the 2',7'-dichlorodihydrofluorescein diacetate (H2DCFDA) assay-limitations of method. Environ Sci Pollut Res Int 23:12246-12252.
