# Supplementary material for: The Role of Ca2+ Imbalance in the Induction of Acute Oxidative Stress and Cytotoxicity in Cultured Rat Cerebellar Granule Cells Challenged with Tetrabromobisphenol A
Source: Neurochem Res. 2016 Oct 7;42(3):777–87. doi: 10.1007/s11064-016-2075-x (PMC5357503; doi:10.1007/s11064-016-2075-x)
Supplement: Supplementary file 2 — Supplementary material 2 (PDF 436 KB) [file 11064_2016_2075_MOESM2_ESM.pdf]

Online resource 2

Supplementary material to the Neurochemical Research article: "The role of Ca^{2+} imbalance in the induction of acute oxidative stress and cytotoxicity in cultured rat cerebellar granule cells challenged with tetrabromobisphenol A"

Authors: Elzbieta Zieminska, Jacek Lenart, Dominik Diamandakis, and Jerzy W. Lazarewicz

Affiliation: Department of Neurochemistry, Mossakowski Medical Research Centre, Polish Academy of Sciences, Warsaw, Poland

E-mail of the corresponding author: jerzyl@imdik.pan.pl

Effects of TBBPA on intracellular Ca^{2+} level and ROS production in primary CGC cultures measured with confocal fluorescence microscopy: concentration-effect relationship and modulation by NMDAR and RyR antagonists.

In the main article a standard fluorescence plate reader was used to measure changes in fluorescence of the Ca^{2+}-sensitive and ROS-sensitive fluorescent probes in CGC cultures challenged with TBBPA. However, there are arguments that standard plate reader-based methods are not sufficiently sensitive and reliable to measure the highly dynamic and transient changes in $[Ca^{2+}]_i$ that occur during chemical exposure [1], and these reservations may also apply to measurements using the other fluorescent probes. Although the results concerning TBBPA-induced changes in $[Ca^{2+}]_i$ presented in the main article are consistent with our previously published data based on confocal microscopy measurements [2], we decided it prudent to supplement the current plate-reader measurements with confocal microscopy measurements, especially since our DCF fluorescence results had not been confirmed previously using fluorescence microscopy.

In the experiment presented in this supplementary material the CGC cultures were prepared, cultured and treated exactly as is described in the main article. The only difference

was the use of the confocal microscope to measure the fluorescence. The fluorescence at 530 nm of fluo-3 and DCF was measured every 30 sec using an LSM 510 confocal microscope equipped with a 488-nm argon laser. Online data acquisition was via LSM 510 version 3.2 (Carl Zeiss AG, Jena, Germany). The data in Fig. 1 and Fig. 2 were obtained in one example experiment, using the same preparation of CGC cultures. Four wells were used per one treatment. The results from each well consisted of the average data collected from 15 randomly selected objects: cell bodies or their conglomerates. The corresponding data from 4 wells representing the same treatment were combined and presented as the mean ± SD.

Fig. 1A shows approximately two- and three-fold increases in the fluo-3 fluorescence induced in CGC by 10 μM and 25 μM TBBPA, respectively; this reflects a TBBPA concentration-dependent increases in $[Ca^{2+}]_i$. Fig. 1B shows that the application of the combination of NMDAR and RyR antagonists, 0.5 μM MK-801 and 200 μM ryanodine with 2.5 μM bastadin 12, suppressed the increases in $[Ca^{2+}]_i$ induced by TBBPA at both concentrations. An increase in $[Ca^{2+}]_i$ evoked by 10 μM TBBPA, measured after 10 min, was reduced from 183% to 76% of the control level, and the effect of 25 μM TBBPA on $[Ca^{2+}]_i$ decreased from 234% to 124%. Application of these antagonists to control and/or DMSO-treated CGC had only negligible or very transient (ryanodine) effects on fluo-3 fluorescence (results not shown).

Fig. 2A displays increases in DCF fluorescence in CGC challenged with 10 and 25 μM TBBPA, reflecting enhanced production of ROS that was dependent on TBBPA concentration. This effect was completely eliminated in the presence of 0.5 μM MK-801 and 200 μM ryanodine with 2.5 μM bastadin 12 (Fig. 2B).

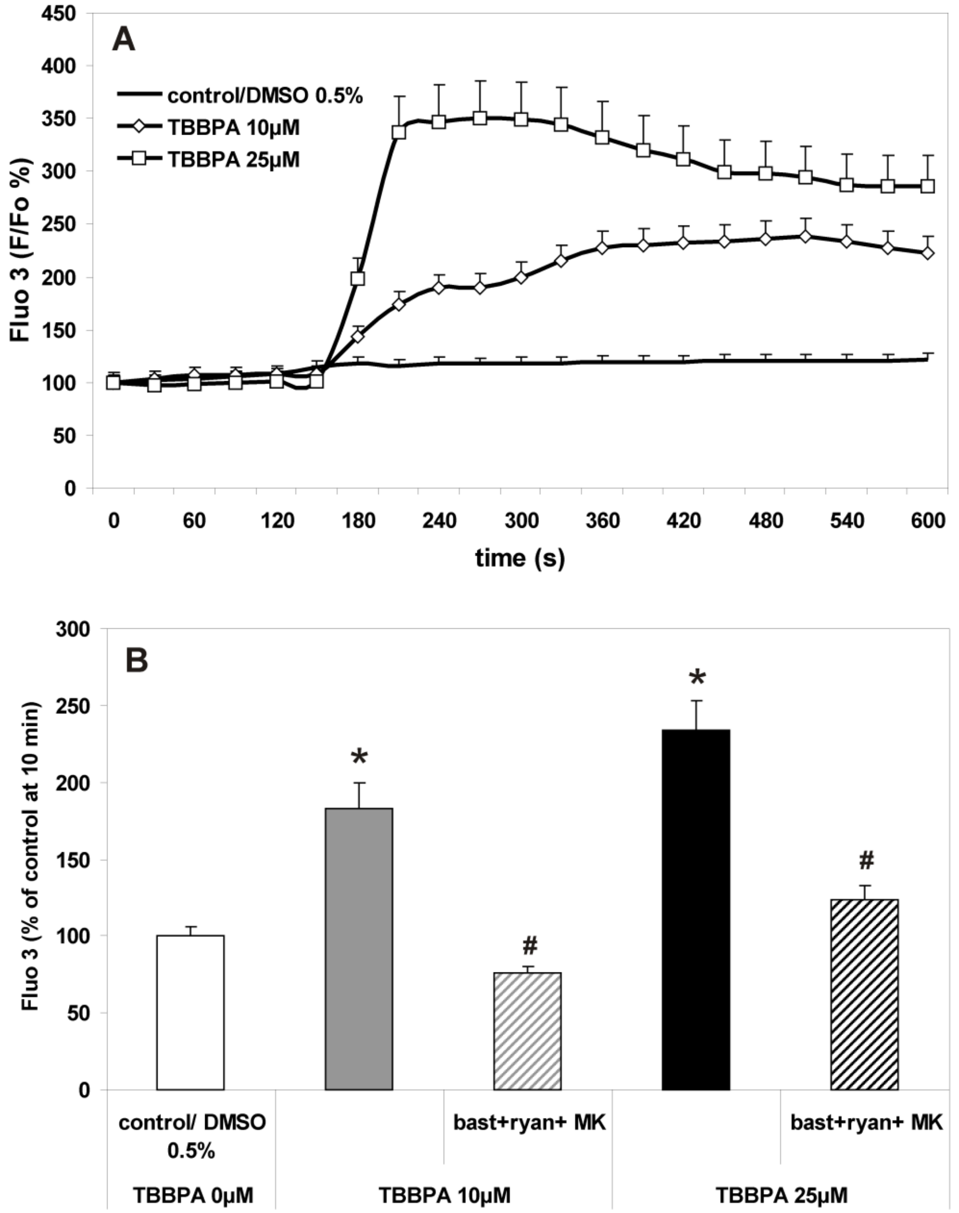

Fig. 1 TBBPA-induced increases in intracellular Ca^{2+} concentration in primary CGC cultures: the fluorescence of the Ca^{2+}-sensitive probe fluo-3 was measured with confocal fluorescence microscopy. **A** The concentration-dependent effect of 10 and 25 µM TBBPA vs. vehicle (0.5% DMSO) on the fluorescence of fluo-3. **B** Inhibition of the effect of 10 and 25 µM TBBPA by the combination of RyR and NMDAR antagonists 2.5 µM bastadin 12 (bast), 200 µM ryanodine (ryan) and 0.5 µM MK-801 (MK). The results are from one example experiment (the mean values ± SD from 4 repetitions). *Results significantly different from the control. #Results significantly different from the corresponding group treated only with TBBPA ($p<0.05$)

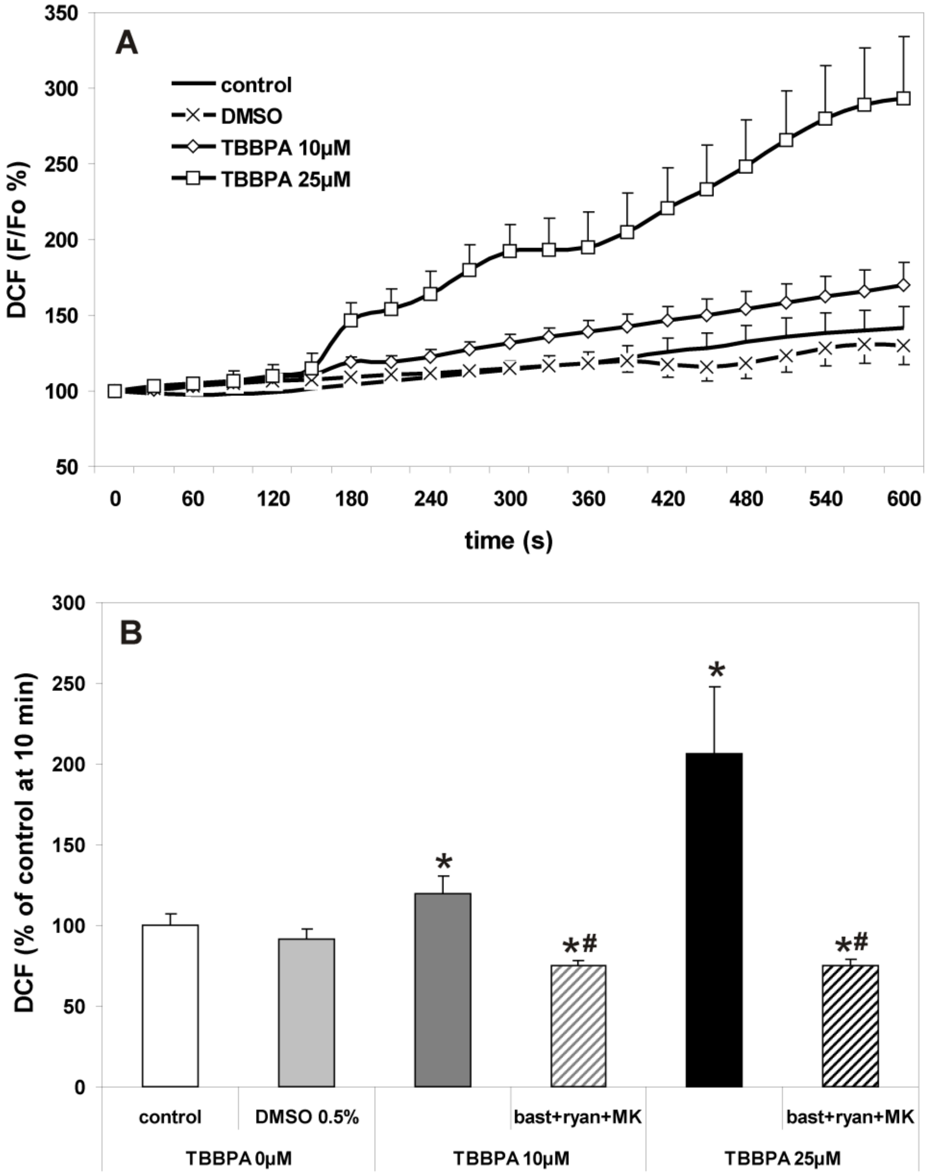

Fig. 2 Increase in ROS production in primary cultures of CGC treated with TBBPA: the fluorescence of DCF was measured with confocal fluorescence microscopy. **A** The concentration-dependent effects of 10 and 25 µM TBBPA vs. vehicle (0.5% DMSO) on the fluorescence of DCF. **B** Modulation of the effects of 10 and 25 µM TBBPA by the combination of RyR and NMDAR antagonists 2.5 µM bastadin 12 (bast), 200 µM ryanodine (ryan) and 0.5 µM MK-801 (MK). The results are from one example experiment (the mean values ± SD from four repetitions). [*]Results significantly different from the control. [#]Results significantly different from the corresponding group treated only with TBBPA ($p<0.05$)

Data measured with the fluorescence plate reader are known to show a step-wise increases in the baseline fluorescence of the calcium-sensitive probes, whereas the single-cell fluorescence microscopy shows a stable basal level of fluorescence [1]. This effect may reflect, amongst other things, the leakage of the fluorescence probe to the Ca^{2+}-reach incubation medium. The increase in $[Ca^{2+}]_i$ evoked by TBBPA in PC12 cells has been shown using fluorescence microscopy to be fast and transient, whereas in the plate reader it was step-wise and persistent [1]. Our data confirm these differences in the stability of the baseline intracellular calcium level depending on the method of measurement used (compare Fig. 1 of the main article with Fig. 1 of this supplementary material). In the present data, unlike in those experiments using PC12 cells [1], we did not observe the full recovery of the low baseline $[Ca^{2+}]_i$ level after its rapid TBBPA-evoked increase, nevertheless, a tendency towards a decrease in fluo-3 fluorescence is visible (this supplementary material, Fig. 1). This difference in the dynamics of TBBPA-evoked changes in $[Ca^{2+}]_i$ in PC12 and CGC may be a reflection of different mechanisms of TBBPA-evoked increases in $[Ca^{2+}]_i$ in these cell models.

The persistent, step-wise increase in both the basal and TBBPA-evoked DCF fluorescence was observed in both the plate reader and confocal microscope measurements (Figs 2 of the main article and of this supplementary material). According to our interpretation, these fluorescence measurements reflect a constant physiological production of ROS in CGC and in addition bursts of production of ROS that are induced by TBBPA. Consequently, both of these mechanisms have an influence on the dynamics of the changes in DCF fluorescence in cells that are at rest and exposed to TBBPA.

Our results show that increases in fluo-3 and DCF fluorescence in TBBPA-challenged CGC are dependent on TBBPA concentration and that this increase can be eliminated by

NMDAR and RyR antagonists. The results from confocal fluorescent microscopy, are consistent with data obtained using a fluorescence plate reader (Fig. 1 and Fig. 2 of the main article). In addition, the results presented here concerning the effects of TBBPA on fluo-3 fluorescence and the modulation of these effects by NMDAR and RyR antagonists are consistent with our previously published data, also obtained using confocal fluorescence microscopy [2]. Collectively, the whole suite of results presented both in the main article and our supplementary information support the conclusion of the main article, that is, in CGC an increase in $[Ca^{2+}]_i$ induced by 10 μM TBBPA is a primary event triggering ROS production and oxidative stress.

References

[1] Meijer M, Hendriks HS, Heusinkveld HJ, Langeveld WT, Westerink RH (2014) Comparison of plate reader-based methods with fluorescence microscopy for measurements of intracellular calcium levels for the assessment of in vitro neurotoxicity. Neurotoxicology 45:31-37.

[2] Zieminska E, Stafiej A, Toczylowska B, Albrecht J, Lazarewicz JW (2015) Role of ryanodine and NMDA receptors in tetrabromobisphenol A-induced calcium imbalance and cytotoxicity in primary cultures of rat cerebellar granule cells. Neurotox Res 28:195-208.
